# Effects of predation environment and food availability on somatic growth in the Livebearing Fish *Brachyrhaphis rhabdophora* (Pisces: Poeciliidae)

**DOI:** 10.1002/ece3.459

**Published:** 2013-01-07

**Authors:** Brittany H Gale, Jerald B Johnson, G Bruce Schaalje, Mark C Belk

**Affiliations:** 1 Department of Biology, Brigham Young University401 WIDB, Provo, UT, 84602; 2 Evolutionary Ecology Laboratories, Brigham Young UniversityProvo, UT, 84602; 3 Monte L. Bean Life Science Museum, Brigham Young UniversityProvo, UT, 84602; 4 Department of Statistics, Brigham Young University223 TMCB, Provo, UT, 84602

**Keywords:** *Brachyrhaphis rhabdophora*, crossing reaction norm, food availability, growth rate, predation

## Abstract

Variation in somatic growth rates is of great interest to biologists because of the relationship between growth and other fitness-determining traits, and it results from both genetic and environmentally induced variation (i.e. plasticity). Theoretical predictions suggest that mean somatic growth rates and the shape of the reaction norm for growth can be influenced by variation in predator-induced mortality rates. Few studies have focused on variation in reaction norms for growth in response to resource availability between high-predation and low-predation environments. We used juvenile *Brachyrhaphis rhabdophora* from high-predation and low-predation environments to test for variation in mean growth rates and for variation in reaction norms for growth at two levels of food availability in a common-environment experiment. To test for variation in growth rates in the field, we compared somatic growth rates in juveniles in high-predation and low-predation environments. In the common-environment experiment, mean growth rates did not differ between fish from differing predation environments, but the interaction between predation environment and food level took the form of a crossing reaction norm for both growth in length and mass. Fish from low-predation environments exhibited no significant difference in growth rate between high and low food treatments. In contrast, fish from high-predation environments exhibited variation in growth rates between high and low food treatments, with higher food availability resulting in higher growth rates. In the field, individuals in the high-predation environment grow at a faster rate than those in low-predation environments at the smallest sizes (comparable to sizes in the common-environment experiment). These data provide no evidence for evolved differences in mean growth rates between predation environments. However, fish from high-predation environments exhibited greater plasticity in growth rates in response to resource availability suggesting that predation environments may exhibit increased variation in food availability for prey fish and consequent selection for plasticity.

## Introduction

Variation in somatic growth rates has interested biologists for decades because of the relationship between growth and other fitness-determining traits (i.e. fecundity, survival, and body size; Arendt and Wilson 1999; Conover and Munch 2002; Olsen et al. 2004; Birkeland and Dayton 2005). Observed variation in growth rates among populations in many species is due in part to underlying additive genetic variation as evidenced by the success of artificial selection on growth rates in domesticated plants and animals (Price 1984; Yamasaki et al. 2007; Biro and Post 2008; Careau et al. 2010). However, in many organisms, growth rates are also responsive to variation in the environment and as such show strong phenotypic plasticity (Gotthard et al. 1994; Conover and Schultz 1995; Belk et al. 2005; Conover et al. 2009; Forero-Montana et al. 2010; Liao et al. 2010). Realized growth rates result from the interaction between variation from additive genetic sources and variation in response to environmental variation.

Selection can act not only on variation in mean differences in growth rates, but also on variation in the shape or orientation of the reaction norm for growth across contrasting environments. Thus, the shape of the reaction norm can be molded by natural selection to improve performance under environmental conditions experienced by the population (Pigliucci 2001; Dewitt and Scheiner 2004).

Predation can be a strong selective agent on a wide variety of fitness-related traits in prey species (Reznick and Endler 1982; Johnson and Belk 2001; Reznick et al. 2001; Johnson and Basolo 2003; Langerhans et al. 2004; Johnson and Zuniga-Vega 2009). Theoretical predictions suggest variation in predator-induced mortality rates can influence mean growth rates and the shape of the reaction norm for growth (Arendt 1997). The adaptive growth hypothesis predicts that mean growth rates evolve in response to variation in mortality rates at different body sizes (Arendt 1997). If mortality rates decrease at larger body sizes, then accelerated growth rates should evolve in smaller size classes. Conversely, if mortality rates increase as body size increases, there should be no selection for rapid growth rates (Arendt 1997; Arendt and Wilson 1999). Thus, mean growth rates might be expected to differ between high-predation environments and low-predation environments depending on the pattern of size-selective predation. Predation environment may also affect the shape of the reaction norm for growth across levels of resource availability (Grether et al. 2001; Zandona et al. 2011). High variance in resource availability over short time scales can select for high levels of plasticity in growth (i.e., a resource-sensitive reaction norm; Nylin and Gotthard 1998). Less variability in resource availability may result in a relatively flat (i.e., insensitive), and nonplastic reaction norm for growth (Nylin 1992; Stearns and Kawecki 1994). If predators increase the variability in available resources either by altering prey behavior or habitat use (Fraser and Gilliam 1987; Fraser et al. 2004), then selection could act to increase the sensitivity of the reaction norm to resource availability (i.e. a strong plastic effect in growth rates depending on resource availability; Billman et al. 2011; Bolnick and Preisser 2005; Grether et al. 2001).

Relatively few studies have focused on adaptive growth rates in response to different predation environments, and most such studies test only for differences in mean growth rates rather than differences in reaction norms. Growth rate responses varied from no detectable difference in mean growth rate between contrasting environments (*Brachyrhaphis rhabdophora*; Johnson, 2001; bluegill sunfish *Lepomis macrochirus*; Belk, 1995) to higher mean growth rate in high-predation environments (Utah chub, *Gila atraria*; Johnson and Belk, 1999; bluegill sunfish, *L. macrochirus*; Belk and Hales, 1993). However, a more complete study on female Trinidadian guppies (*Poecilia reticulata*) showed a variety of responses in mean growth rates and reaction norms among high- and low-predation environments (Arendt and Reznick 2005). Arendt and Reznick (2005) suggested that growth rates in guppies are locally adapted to resource availability rather than to the direct effect of predation. To determine the generality of growth responses to predation environments and resource availability, it is important to test for differences in growth rates in independently evolved systems with similar selective regimes.

**Figure 1:**
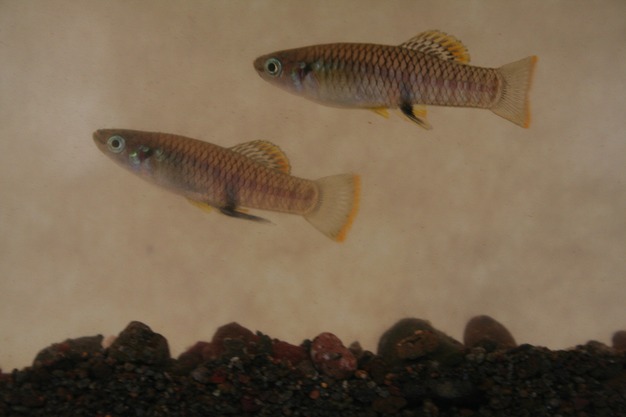
Male (bottom) and female (top) *Brachyrhaphis rhabdophora*. Photograph by M. C. Belk.

*Brachyrhaphis rhabdophora*, a neotropical livebearing fish (Poeciliidae; Fig. 1), provides an excellent comparative system to the Trinidadian guppy. *Brachyrhaphis rhabdophora* occurs in numerous streams and rivers throughout much of the Guanacaste region of Costa Rica, in both high-predation and low-predation environments (Johnson and Belk 2001), and mortality rates differ dramatically between these two types of environments (Johnson and Zuniga-Vega 2009). In addition to the effects of mortality rate, food availability may vary seasonally in coordination with wet and dry seasons (Winemiller 1993; Jennions et al. 2006) and between high- and low-predation environments (Johnson 2002).

Variation in mortality rates and resource availability among populations provides conditions that may select for variation in mean growth rates or reaction norms for growth (i.e. plasticity). We used juvenile *B. rhabdophora* from high-predation and low-predation environments to test for variation in mean growth rates and for variation in reaction norms for growth at two levels of food availability in a common environment, and we compared somatic growth rates of juveniles in the wild from the same two locations (high-predation and low-predation environments).

## Materials and Methods

### Effects of predation environment and food availability: common-environment experiment

To determine the effect of predation environment, food availability, and their interaction on growth rates, we conducted a common-environment experiment (Rader et al. 2005) using juvenile, second-generation, lab-raised (F2) *B. rhabdophora*, from 11 different families. The common-environment experiment followed a split-brood design, where whole units were families, and subunits were individual fish. In this common-environment experiment, F2 juveniles from both a high-predation and a low-predation environment (*n* = 66, Rio Javilla, and *n* = 67, Quebrada Grande, respectively, for specific map locations of these river systems see Johnson and Belk 2001) were randomly assigned and raised at two different levels of food availability (treatments were within families). Using this design, we were able to quantify the effect of predation environment, food availability, and their interaction on growth rate.

To avoid mortality of newly born individuals from handling stress, we used measures of beginning length and mass of newborn individuals that were not used in the experiment, but were from the same locations (Rio Javilla and Quebrada Grande) to determine the average size at birth for fish from each location. Newborn individuals from Quebrada Grande (the low-predation site) averaged 8.50 mm ± 0.47 SD in standard length and 0.012 g ± 0.002 SD in wet mass, *n* = 52. Newborn individuals from Rio Javilla (high-predation site) averaged 7.74 mm ± 0.30 SD in standard length and 0.008 g ± 0.001 SD in wet mass, *n* = 21. Fish from the low-predation environment (Quebrada Grande) were significantly longer and heavier at birth compared to fish from the high-predation environment (Rio Javilla) (*t* = 6.85, df = 71, *P* < 0.001; *t* = 871, df = 71, *P* < 0.001). The clear difference in size at birth between locations and the relatively low variation in size at birth as evidenced by the SD suggests that our results are not biased by use of these values as average starting size for individuals in the experiment. Fish were placed in individual housing cups on day of birth and randomly assigned to either a high or low food treatment. The high food treatment was 15% of somatic mass per day (5% fed at three times per day) and the low food treatment was 3% of somatic mass fed once daily in accordance with methods in Reznick (1983). To compensate for growth, food amounts were increased weekly based on the average estimated growth rate from a pilot study. Fish fed on the low food treatment consumed all food given. Fish fed on the high food treatment generally did not consume all food; hence, a food amount of 15% of somatic mass per day was comparable to an ad libitum feeding regime.

Fish were fed Wardley Small Fry Liquid Food for the first 2 weeks (14 days) and were fed finely ground TetraMin Flakes for the remainder of the time (26 days). The experiment ran for 40 days. *Brachyrhaphis rhabdophora* can mature in as little as 87 days (Johnson 2001); hence, the 40-day experimental period avoided confounding of growth and reproduction. At the end of 40 days, wet masses and standard lengths of each fish were recorded. We assumed prematuration growth of both males and females would be similar, as it is impossible to differentiate between putative males or females before about 35 days (Johnson 2001).

To create a common-environment for the experiment, two large tubs were set up that held the individual housing cups. Housing cups (0.95 L) were randomly assigned to a tub and a location in the support device (maintaining all cups at approximately 0.5 m from the bottom of the tub). The housing cups were opaque to avoid visual cues among experimental individuals. Each tub was equipped with a submersible water heater and a water pump to help maintain uniformity in water temperature throughout individual tubs and between tubs. The temperature in the tubs was maintained at 29°C ± 0.3 such that there were no differences in mean temperature between tubs or through time. This temperature was selected because it is in the middle of the range of temperatures in which *B. rhabdophora* are found in their natural environment (Bussing 1998), and was a temperature at which they reproduced successfully and consistently in the lab (B. Gale, unpubl. data). We followed guidelines from the Institutional Animal Care and Use Committee (BYU IACUC) for maintenance of individuals in the experiment.

To analyze the data from the common-environment experiment, we first determined growth over the course of the experiment by subtracting the ending size measurements from the average beginning size measurements (depending on population of origin) for each individual fish. Response variables were growth in length (natural log transform of gain in standard length) and growth in mass (natural log transform of gain in wet somatic mass). We used a mixed model analysis (Proc MIXED, Littell et al., 1996) with food availability and location of origin (high-predation or low-predation environments) as predictor variables. The interaction between location of origin and food level was also included in the model. Families (i.e., sibling groups) were treated as a random blocking effect. We examined the residuals and removed four fish with extremely large residuals from the analysis (two from high-predation environments and two from low-predation environments).

### Effects of predation environment: field study

To determine the effects of predation environment on growth rates of juvenile *B. rhabdophora* in the field, we used a serial mark-recapture design over a 4-week period during the late dry season (January–February). For a complete description of the mark-recapture study, refer to Appendix A of Johnson and Zuniga-Vega (2009). We calculated growth rates for the juvenile size classes from a high-predation (*n* = 41, Rio Javilla) and a low-predation (*n* = 129, Quebrada Grande) location. Average individual growth rate was calculated by taking the difference between standard length at first capture and standard length at last capture for each individual fish divided by the number of days that had transpired between the initial capture and recapture dates.

To analyze the data from the serial mark-recapture field work, we used a general linear model analysis. The response variable was growth in mm/day, predation environment was the predictor variable, and the covariate was beginning standard length. We modeled the effect of the covariate with a smoothing spline (SAS PROC GLIMMIX, SAS Institute Inc., Cary, NC). We examined the residuals and found no outliers or deviations from assumptions of normality or equal variances; hence, data were not transformed. To test for a difference between locations in the effect of the covariate (beginning standard length), we compared a model with one spline (i.e. no effect of predation environment on relationship between growth and beginning standard length) to a model with two splines (i.e. different relationship between growth and beginning standard length for each predation environment).

## Results

### Effects of predation environment and food availability; from the common-environment experiment

**Figure 2:**
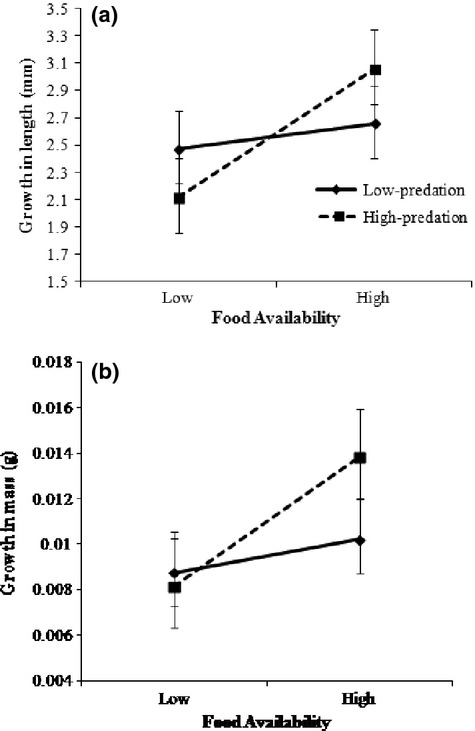
(a) Least-squares means (±1 standard error) of growth in length of juvenile *Brachyrhaphis rhabdophora* from high-predation (Javilla) and low-predation (Grande) environments at low and high levels of food availability in a common-environment experiment. (b) Least-squares means (±1 standard error) of growth in wet mass of juvenile *B. rhabdophora* from high-predation (Javilla) and low-predation (Grande) environments at low and high levels of food availability in a common-environment experiment.

Growth in length did not differ between predation environments, but was affected by food availability; there was also a significant interaction between predation environment and food availability (Table 1). Similarly, growth in mass did not differ between predation environments, but was affected by food availability and by the interaction between predation environment and food availability (Table 1). The interaction between predation environment and food availability took the form of a crossing reaction norm for both growth in length and mass. Fish from low-predation environments exhibited no significant difference in growth rate between high and low food availability treatments (pairwise *t*-test, both *P* > 0.4). In contrast, fish from high-predation environments exhibited significantly higher growth rates in high food availability treatments compared to low food availability (pairwise *t*-test, both *P* < 0.002; Fig. 2).

**Table 1:** Mixed model analysis of covariance results for growth in standard length and wet mass of *Brachyrhaphis rhabdophora* in a common-environment experiment

Response variable	Source of variation	Degrees of freedom (num/den)^1^	*F*-value	*P*-value
Standard length	Predation	1/10.4	0	0.9554
	Food amount	1/118	25.99	<0.0001
	Predation× food amount	1/118	11.58	0.0009
Wet mass	Predation	1/10.7	0.27	0.6151
	Food amount	1/120	17.01	<0.0001
	Predation× food amount	1/120	5.23	0.024

1 Fractional degrees of freedom are due to the Kenward–Roger adjustment (Kenward and Roger 1997).

### Effects of predation environment; from the field

**Figure 3:**
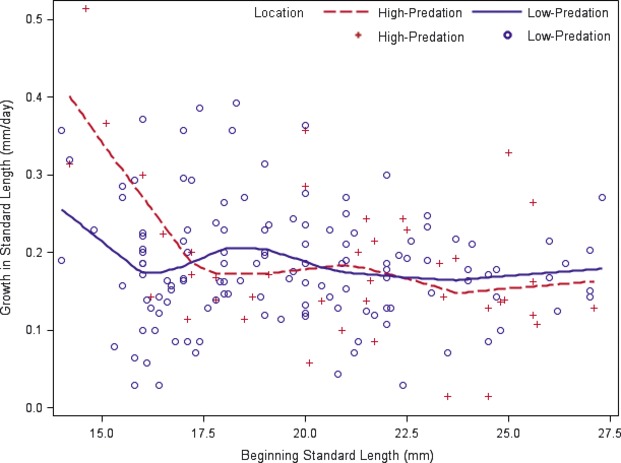
Growth in standard length (mm/day) of juvenile *Brachyrhaphis rhabdophora* from high-predation (Javilla) and low-predation (Grande) environments plotted against beginning standard length in the field mark-recapture experiment. Lines are smoothed splines (SAS PROC GLIMMIX) representing the nonlinear relationship between growth and beginning standard length. Splines for the two locations were significantly different (χ^2^ = 10.12, df = 4, *P* < 0.05).

The model with two splines fit significantly better than the model with one spline (χ^2^ = 10.12, df = 4, *P* < 0.05), indicating that the relationship between growth rate and beginning standard length differed between high- and low-predation environments. Smaller juveniles grow faster than larger juveniles in both locations. At smaller sizes (comparable to sizes tested in the common-environment experiment), individuals from the high-predation environment grow at a faster rate than those from a low-predation environment (Fig. 3).

## Discussion

Mean growth rates of juvenile *B. rhabdophora* do not differ in the common-environment experiment, but the reaction norms of response to food availability (i.e. the plastic response) differ between high-predation and low-predation environments. Growth rates change in response to variation in food availability in high-predation environments, but not in low-predation environments. Plasticity can evolve in response to variation in the environment that occurs over a temporal scale that is shorter than the expected lifetime (Thompson 1991; Relyea 2002; Dewitt and Scheiner 2004; Orizaola et al. 2012). Food availability may vary seasonally with wet and dry seasons (Winemiller 1993; Grether et al. 2001; Jennions et al. 2006). Although *B. rhabdophora* can live 2–3 years in the laboratory, fish in the field are unlikely to live that long (Johnson and Zuniga-Vega 2009); hence, plastic responses to food availability may be unlikely based only on possible seasonal variation.

Why would plasticity in response to food availability evolve in high-predation environments and not in low-predation environments? One possibility is that the direct and indirect effects of predation may enhance variability in food resources between the two habitat types (Arendt 1997; Luttbeg et al. 2003; Bolnick and Preisser 2005). Prey responds behaviorally to the presence of predators and balance feeding activities with the risk of predation (Lima and Dill 1990; Stamps 2007; Tirok and Gaedke 2010). Variation in risk of predation could drive variation in food availability for prey and consequent selection for plasticity (Fraser and Gilliam 1987; Sih 1992; Relyea 2002; Dernekbasi et al. 2010).

We found no evidence for genetically based differences in mean growth rate between fish from high-predation and low-predation environments (a pattern previously documented for male *B. rhabdophora*; Johnson 2001). However, we did observe differences in mean growth rate between fish from high-predation and low-predation environments in the field at the smallest juvenile stage (Fig. 3). The only way to reconcile these two outcomes given the results of the common-environment experiment is to ascribe the difference in growth rate observed in the field to environmental effects or to a genetic by environment (GXE) interaction. If resources are more available for the smallest size classes in high-predation environments compared to low-predation environments, we would expect to see higher growth rates in high-predation environments consistent with our observation (Arendt and Reznick 2005). High-predation environments could have higher resources because of lower densities of conspecifics or because of general characteristics of high-predation environments, such as lower canopy cover and higher resultant primary production (Grether et al. 2001; Reznick et al. 2001; Johnson 2002; Bolnick and Preisser 2005; Zandona et al. 2011).

In the wild at smaller sizes growth rates are higher in high-predation environments. However, as size increases, growth rates decline in the high-predation environment such that larger fish have similar growth rates in both environments. This is consistent with the adaptive growth hypothesis that predicts that if mortality rates increase at larger sizes then selection should act to decrease growth rate and prolong the time spent in the smaller size class. In high-predation environments mortality rates increase with size especially as individuals approach the largest size class (Johnson and Zuniga-Vega 2009). From the perspective of the adaptive growth hypothesis, fish in high-predation environments should grow rapidly early to minimize the time to reproductive maturity, and then grow slowly thereafter to decrease the probability or increase the time to transitioning into the largest size class with the highest mortality rate (Conover and Present 1990; Arendt 1997; Arendt and Wilson 1999). Fish in the low-predation environment face no such selective effects from the mortality schedule. In the low-predation environment mortality rates decrease with size especially in the transition to the largest size class (Johnson and Zuniga-Vega 2009). Hence, in low-predation environments selection may be stronger on body size directly (for gains in fecundity, etc.) rather than on growth rate and how quickly body size may be attained (Creighton et al. 2009; Johnson and Zuniga-Vega 2009). In high-predation environments, growth in the juvenile life stages is an important contributor to overall population dynamics; whereas, growth in early stages is not important to population dynamics in the low-predation environments. Thus, selection for increased growth rates may occur in high-predation environments and not in low-predation environments. Because of the potential differences in the variability in resource availability, the ability to use resources efficiently may be under selection in high-predation environments and not in low-predation environments (Johnson and Zuniga-Vega 2009).

In a comparable study on Trinidadian guppies, Arendt and Reznick (2005) found variation in patterns of growth rate evolution among populations. At two sets of paired locations, growth rates were higher in high-predation environments at both high and low food levels compared to low-predation environments. However, at two other sets of paired locations, there were no significant differences in growth rates between high- and low-predation environments at high or low food levels. In an introduction experiment, growth rates evolved to the form of a crossing reaction norm, such that fish from the high-predation environment exhibited higher growth rates at the high food level compared to fish from the low-predation environment, but growth rates were equal at the low food level. These authors conclude that when growth rates evolve differentially between high- and low-predation environments, the result is generally faster growth in high-predation (and generally high resource availability) environments, and thus growth rates seem to be an adaptation to resource availability rather than direct effects of predation (Arendt and Reznick 2005). Our results for *B. rhabdophora* differ somewhat in that mean growth rates did not differ between high- and low-predation environments, but there was a significant crossing reaction norm for growth at different resource levels between predation environments. In the low-predation environment, fish grew at the same rate on high and low food levels; however, fish from the high-predation environment showed significantly higher growth on high food levels compared to low food levels. Although a crossing reaction norm for growth was observed in Trinidadian guppies between high- and low-predation environments in the introduction experiment, the pattern is somewhat different from the crossing reaction norm observed in *B. rhabdophora*. In Trinidadian guppies, both high- and low-predation populations responded to high food levels with increased growth rates, but fish from the high-predation environment showed a significantly greater response (Arendt and Reznick 2005). In contrast, in *B. rhabdophora*, it was only the fish from the high-predation environment that exhibited significantly increased growth rates in response to high food levels. The pattern of growth in *B. rhabdophora* from the low-predation environment is consistent with growth patterns seen in response to chronically low food levels (pessimistic growth, Iwasa 1991). Such patterns were not observed in Trinidadian guppies (Arendt and Reznick 2005). Patterns observed among populations of *B. rhabdophora* suggest adaptation to variation in resource availability in high-predation environments and adaptation to chronically low food levels in low-predation environments. Additional studies on other comparable systems using consistent methods and life stages should be conducted to help differentiate effects of predation environment and resource availability on the evolution of growth.

In summary, we found no differences in mean growth between high-predation and low- predation populations reared under common environmental conditions. However, there was an important interaction between predation environment and food availability – fish from high-predation environments were responsive to variation in food availability, whereas fish from low-predation environments were not. This change in phenotypic plasticity shown by the crossing reaction norm between population types (predation environment) indicates an evolved difference in growth pattern between predation environments in *B. rhabdophora*. These results suggest that predation environment can selectively influence the shape of the reaction norm for growth across levels of resource availability, in addition to affecting mean growth rates of prey.
